# Splenic Stiffness Measurement Combined With Liver Stiffness Measurement Compared With Baveno VII Criteria in Predicting the Presence of Oesophageal and Gastric Varices in Patients With Compensated Advanced Liver Cell Disease (cALCD)

**DOI:** 10.7759/cureus.65954

**Published:** 2024-08-01

**Authors:** Arjuna P de Silva, Madunil A Niriella, Nilanga Nishad, Hishali Jayasundara, Hiruni Jayasena, Vajira T Samarawickrama, Chamila Ranawaka, Kumarini Basnayake, Shamila T de Silva, Hithanadura J de Silva

**Affiliations:** 1 Department of Medicine, University Medical Unit, Colombo North Teaching Hospital, Faculty of Medicine, University of Kelaniya, Ragama, LKA; 2 Gastroenterology and Hepatology, Addenbrooke's Hospital, Cambridge University Hospitals National Health Service Foundation Trust, Cambridge, GBR; 3 Department of Gastroenterology, University Medical Unit, Colombo North Teaching Hospital, Faculty of Medicine, University of Kelaniya, Ragama, LKA; 4 Department of Medicine, Faculty of Medicine, General Sir John Kotelawala Defence University, Colombo, LKA; 5 Department of Gastroenterology and Hepatology, University Medical Unit, Colombo North Teaching Hospital, Faculty of Medicine, University of Kelaniya, Ragama, LKA; 6 Gastroenterology, Cambridge University Hospitals National Health Service Foundation Trust, Cambridge, GBR; 7 Department of Medicine, Faculty of Medicine, University of Kelaniya, Ragama, LKA

**Keywords:** fibrosis, liver cirrhosis, portal hypertension, splenic stiffness, liver stiffness measurement, oesophageal varices

## Abstract

Introduction: Liver stiffness measurement (LSM) using vibration-controlled transient elastography (VCTE) is being increasingly used as a screening tool to predict varices. Our aim was to test the utility of Baveno VII criteria and other combinations of LSM, platelet count (PC), and splenic stiffness measurement (SSM) to predict the presence of varices in a cohort of Sri Lankan patients with compensated advanced liver cell disease (cALCD).

Methods: Consecutive patients with newly diagnosed Child-Pugh class A cALCD (non-viral, BMI<30) were recruited prospectively. They underwent gastroscopy. LSM and SSM were taken using vibration-controlled transient elastography (VCTE) (Echosens FibroScan 502 Touch; Echosens SA, Paris, France) by a single operator who was unaware of endoscopy findings. Sensitivity, specificity, positive predictive value (PPV), negative predictive value (NPV), and accuracy of different Baveno VII criteria to predict the varices and different combinations of LSM, SSM, and PC were also explored.

Results: One hundred and seventy-four individuals were recruited. The mean age was 61.4 ((95% CI: 59.7-62.8) years. A total of 110 individuals were males, and 106 had varices. Our results indicated that the three Baveno VII criteria had sensitivities of 61%, 63%, and 42%, and specificities of 79%, 77%, and 87% to predict varices. SSM>30kPa alone and in combination with LSM>15kPa had sensitivities of 81 and 75%, specificities of 72 and 83%, PPVs of 82 and 87%, NPVs of 71% and 67%, and accuracies of 78 and 78%, respectively, to predict varices.

Conclusion: Baveno VII criteria had a low sensitivity but high specificity in predicting the presence of varices. However, SSM>30kPa alone or in combination with LSM>15kPa had better sensitivity, specificity, PPV, NPV, and accuracy in predicting varices.

## Introduction

Chronic liver disease is a major cause of morbidity and mortality worldwide. The progression of liver fibrosis to cirrhosis is a complex process that involves a series of events, leading to the development of portal hypertension, which is characterized by an increase in intrahepatic vascular resistance and a decrease in portal blood flow [1]. The gold standard for measuring portal venous pressure is measuring the hepatic portal venous pressure gradient (HPVPG) by catheterizing the portal vein [2,3]. However, this is an invasive procedure, and therefore, non-invasive methods have been developed to identify patients with clinically significant portal hypertension (CSPH). CSPH is defined as an HPVPG of ≥10 mmHg and is associated with an increased risk of development of varices and overt clinical decompensation in the form of variceal hemorrhage, ascites, and hepatic encephalopathy to name a few [2,4]. Early detection of varices is paramount in patients with compensated advanced liver cell disease (cALCD) to prevent bleeding [5]. Endoscopy is the gold standard for the detection of varices, but it is invasive, costly, and requires trained personnel [6]. Thus, non-invasive tests can help avoid unnecessary endoscopy and aid in predicting prognosis as well [7].

Vibration-controlled transient elastography (VCTE) is a non-invasive tool for liver stiffness measurement (LSM) and is used to evaluate liver fibrosis [7,8]. LSM has been shown to correlate well with the degree of fibrosis in patients with chronic liver disease and can predict the development of portal hypertension [9]. The Baveno VII criteria were developed to identify patients with cALCD, who have a low risk of developing varices and therefore can avoid repeated endoscopic screening [10]. CSPH was ruled out if patients had an LSM of 15 kPa or less and a platelet count over 150 × 109 /L [1,11]. CSPH was ruled in if a patient had an LSM of 20-25 kPa with a platelet count of less than 150 × 109 /L or an LSM of more than 25 kPa, irrespective of the platelet count [1,11]. However, there remained an indeterminate area where there could be CSPH with an LSM of 15-19.9 kPa and a platelet count <110 × 109 /L.

Recently, the utility of the Baveno VII criteria alone in predicting varices has been questioned, and alternative non-invasive methods have been proposed [12]. Splenic stiffness measurement (SSM) is another non-invasive method that has been shown to correlate with the presence of portal hypertension and can predict the development of varices [12-13].

We hypothesized that combining LSM and SSM may improve the accuracy of predicting varices. Therefore, the aim of our study was to compare the utility of the Baveno VII criteria and different combinations of LSM, platelet count (PC), and SSM to predict oesophageal and gastric varices in a cohort of Sri Lankan patients with cALCD.

## Materials and methods

Patients

This was a prospective study conducted at the University Medical Unit, Colombo North Teaching Hospital, Ragama, Sri Lanka from January 2019 to January 2021. All patients underwent a detailed history and physical examination. Laboratory investigations including complete blood count, liver function tests, prothrombin time, and viral serology (hepatitis B and C) were performed, and the Child-Turcotte-Pugh (CTP) score was calculated for each patient.

All participants included in the study were patients diagnosed with cALCD following detailed history and physical examination together with the use of biochemical indices (full blood count, liver function tests, prothrombin time) and baseline imaging of the liver using ultrasound abdomen. All diagnosed patients with cALCD were then classified according to CTP score (graded as A, B, or C). Furthermore, all diagnosed patients with cALCD had screening for viral hepatitis (hepatitis B and C) done. Consecutive patients with newly diagnosed cALCD (CTP class A) were recruited for the study. Inclusion criteria were as follows: (1) age ≥ 18 years, (2) non-viral etiology of liver disease, (3) BMI <30 kg/m2, and (4) ability to provide informed consent. Patients with decompensated liver disease (CTP class B or C), viral etiology of liver disease, active alcohol or drug abuse, hepatocellular carcinoma, extrahepatic portal obstruction, or any other cause of portal hypertension and who had splenectomy were excluded. All patients provided written informed consent.

VCTE

LSM

LSMs were performed using transient elastography (Echosens FibroScan 502 Touch; Echosens SA, Paris, France) by a single experienced operator (performed > 1000 scans) who was blinded to the clinical and endoscopic findings. The participants were fasting for more than four hours as per guidelines. We included only the LSMs with ≥ 10 valid measurements with a success rate of ≥ 60%, and/or an interquartile range/median ratio (IQR/M) ≥ 0.3.

SSM

SSM was performed using the same LSM probe, with the study participants placed in a supine position with maximal abduction of the left arm, and the probe was positioned in an intercostal space with maximal splenic dullness on percussion. We included SSMs with ≥ 10 valid measurements. The M probe was used to measure both LSM and SSM. LSM and SSM values were recorded in kilopascals (kPa).

Upper gastrointestinal endoscopy

All patients underwent upper gastrointestinal endoscopy to detect the presence of oesophageal and gastric varices. Endoscopy was performed by experienced endoscopists (performed > 5000 gastroscopies on patients with cirrhosis) who were unaware of the LSM, SSM, and platelet count values. The presence or absence of varices was documented for each participant in the study.

Statistical analysis

The sensitivity, specificity, positive predictive value (PPV), negative predictive value (NPV), and accuracy of different Baveno VII criteria to predict varices were calculated. Different combinations of LSM, SSM, and platelet counts were also explored for their utility in predicting varices. Statistical analysis was performed using IBM SPSS Statistics for Windows, Version 25, (Released 2017; IBM Corp., Armonk, New York, United States). Continuous variables were expressed as mean ± standard deviation (SD). Categorical variables were expressed as numbers and percentages. A p-value of less than 0.05 was considered statistically significant.

## Results

Patient characteristics

One hundred and seventy-four individuals were recruited for the study. The mean age was 61 years and the majority of the participants were males (n=110, 63.2%). Alcohol-related liver disease was the commonest etiology (n=108, 62%) followed by non-alcoholic fatty liver disease (n=33, 19%). The mean BMI was 25.4 kg/m2. The mean LSM was 23.5 kPa, and the mean SSM was 34.6 kPa. One hundred and twenty-six patients were noted to have oesophageal or gastric varices or both on endoscopy. Furthermore, 129 (74%) patients were noted to have splenomegaly (Table 1).

**Table 1:** Basic characteristics of the participants.

Characteristic	n (%) or mean	95% CI
Age, years (mean)	61.4	(59.7-62.8)
Sex, n (%)		
Male	110 (63.2)	(55.8-70.1)
Female	64 (36.8)	(29.9-44.2)
Etiology of liver disease, n (%)		
Alcohol	108 (62.1)	(54.7- 67.9)
Non-alcoholic fatty liver disease	33 (19.0)	(15.8-27.9)
Cryptogenic	33 (19.0)	(15.8-27.9)
BMI, kg/m2 (mean)	25.4	(24.6-26.2)
Child-Pugh Class, n (%)		
Class A	174 (100)	
Platelet count, x10^9^/L (mean)	130.8	(117.6-144.1)
Liver stiffness measurement, kPa (mean)	23.5	(21.7-25.2)
Spleen stiffness measurement, kPa (mean)	34.6	(32.0-37.3)
Oesophageal varices, n (%)	106 (61.1)	(53.5-67.9)
Gastric varices, n (%)	20 (11.5)	(7.6-17.1)
Splenomegaly, n (%)	129 (74.1)	(67.2-80.1)

The mean (SD) platelet count of patients with varices was 123 (54) /L and without varices was 175 (69) /L (p< 0.001). The mean (SD) of “median LSM” value of patients with varices was 35 (18) kPa and without varices was 19(11) (p< 0.001) kPa. The mean (SD) of “median SSM” value of patients with varices was 50 (18) kPa and without varices was 27 (15) kPa (p< 0.001).

Sensitivity, specificity, PPV, NPV, and accuracy of LSM > 25, LSM > 20 and PC < 150, LSM > 15 and PC < 110, SSM > 30 with LSM > 15 and SSM > 30 have been calculated and tabulated in Table 2. SSM > 30 kPa alone or in combination with LSM > 15 kPa had better sensitivity, specificity, PPV, NPV, and accuracy in predicting the presence of oesophageal and gastric varices (Table 2).

**Table 2:** Prediction of varices criteria *Baveno VII criteria LSM: liver stiffness measurement; PC: platelet count; SSM: splenic stiffness measurement

	LSM >25*	LSM >20 and PC <150*	LSM >15 and PC <110*	SSM>30	LSM >15 and SSM>30
Sensitivity	61.30%	63.30%	42.20%	81.40%	74.50%
Specificity	79.40%	77.40%	87.10%	72.30%	82.80%
Positive predictive value	82.30%	80.30%	82.60%	82.20%	87.40%
Negative predictive value	56.80%	59.30%	50.90%	71.20%	67.10%
Accuracy	68.40%	69.10%	60.50%	77.80%	77.70%

## Discussion

Our study compared the utility of the Baveno VII criteria and combinations of LSM, PC, and SSM to predict oesophageal and gastric varices in a cohort of Sri Lankan patients with non-viral cALCD. To the best of our knowledge, this is the first study of its sort conducted in a South Asian cohort to examine this issue. Our findings suggested that SSM > 30 kPa alone or in combination with LSM > 15 kPa had better sensitivity, specificity, PPV, NPV, and accuracy in predicting varices than the Baveno VII criteria alone.

The Baveno VII criteria had high specificity but low sensitivity to predict the presence of varices. Thus, the Baveno VII criteria alone may not identify many patients with CSPH and may not detect those patients with a higher risk of developing varices. This is consistent with previous studies that have questioned the utility of Baveno VII criteria in predicting varices in patients with CLCD [12]. However, it is noteworthy that some studies have found Baveno VII criteria to be accurate in predicting varices even among the subgroups of cirrhosis [14,15].

In contrast, SSM > 30 kPa alone or in combination with LSM > 15 kPa had better sensitivity and negative predictive value to predict varices according to our study findings. This suggests that combining SSM and LSM may further improve the accuracy of predicting varices and in turn reduce the need for unnecessary invasive endoscopic screening [16-18]. Further, this will help with the early initiation of non-selective beta-blocker (NSBB) therapy, which will aid in minimizing and preventing future hepatic decompensation [19].

Platelets are an acute phase reactant and hence a rise in PC can be due to other reasons such as trivial viral infections. Hence removing PC as a variable may also improve the accuracy of results [20]. 

Our study identified the cut-off > 30 kPa for the presence of varices. The cut-off values for SSM in Baveno VII criteria are complex and not clearly established. For viral hepatitis-induced cALCD, it is < 21 kPa to rule out and > 50 kPa to rule in. It is also stated that a cut-off of < 40 kPa can be used in patients who are not candidates for NSBBs (contraindication/intolerance) and in whom endoscopy would be required according to the Baveno VI criteria (LSM by VCTE > 20 kPa or platelet count < 150x109 L), to rule out having to do endoscopy [11,21]. In addition, it is stated that more research is needed to establish cut-offs for cALCD in non-alcoholic fatty liver disease (NAFLD) [6]. Another study gave a cut-off of > 40 kPa for the presence of varices [22].

Our findings are consistent with previous studies that have shown the utility of SSM in predicting the presence of varices in patients with chronic liver disease [14,23].

There are however several limitations in our study. One is that it was conducted in a single center and included a relatively small number of patients. It is also possible that our results may be valid only for cALCD patients with a BMI < 30. Further, we used the Fibroscan 502 and not the Fibroscan Expert 630 (Echosens SA, Paris, France) for SSM measurements, as it was not available at our center. However, most of the data available regarding SSM has been generated using the Fibroscan 502 [13,23]. Our study also did not evaluate the cost-effectiveness of different screening strategies, which may be an important consideration in resource-limited settings.

While further studies are needed to confirm our findings in larger cohorts and in different populations, the need to do a combination of LSM and SSM in the same sitting in all patients with cALCD is an important clinical and practical conclusion of our study, as currently, this is not a universal practice [14].

Hepatic venous pressure gradient (HVPG) is the gold standard for the measurement of portal hypertension. However, as a developing country with limited clinical resources, the facilities for HVPG measurements are not readily available in Sri Lanka. Hence we acknowledge the need for further studies using the HVPG measurements together with SSM for early detection of the presence of CSPH or the presence of varices.

## Conclusions

Our study suggests that SSM > 30 kPa alone or in combination with LSM > 15 kPa may be a more accurate non-invasive tool to predict the presence of varices than the Baveno VII criteria in patients with cALCD. However, it needs to be said that further studies are needed to be undertaken to confirm these findings and to evaluate the cost-effectiveness of different screening strategies.

## References

[1] de Franchis R (2015). Expanding consensus in portal hypertension: report of the Baveno VI Consensus Workshop: stratifying risk and individualizing care for portal hypertension. J Hepatol.

[2] Garcia-Tsao G, Abraldes JG, Berzigotti A, Bosch J (2017). Portal hypertensive bleeding in cirrhosis: risk stratification, diagnosis, and management: 2016 practice guidance by the American Association for the Study of Liver Diseases. Hepatology.

[3] McConnell M, Iwakiri Y (2018). Biology of portal hypertension. Hepatol Int.

[4] Leung JC, Loong TC, Pang J, Wei JL, Wong VW (2018). Invasive and non-invasive assessment of portal hypertension. Hepatol Int.

[5] Sanyal AJ, Bosch J, Blei A, Arroyo V (2008). Portal hypertension and its complications. Gastroenterology.

[6] Pena LR, Cox T, Koch AG, Bosch A (2008). Study comparing oesophageal capsule endoscopy versus EGD in the detection of varices. Dig Liver Dis.

[7] (2015). EASL-ALEH clinical practice guidelines: non-invasive tests for evaluation of liver disease severity and prognosis. J Hepatol.

[8] Sasso M, Miette V, Sandrin L, Beaugrand M (2012). The controlled attenuation parameter (CAP): a novel tool for the non-invasive evaluation of steatosis using Fibroscan. Clin Res Hepatol Gastroenterol.

[9] Yang A, Zhu X, Zhang L (2022). Non-invasive evaluation of NAFLD and the contribution of genes: an MRI-PDFF-based cross-sectional study. Hepatol Int.

[10] de Franchis R, Bosch J, Garcia-Tsao G, Reiberger T, Ripoll C (2022). Baveno VII - renewing consensus in portal hypertension. J Hepatol.

[11] Allaire M, Campion B, Demory A (2023). Baveno VI and VII criteria are not suitable for screening for large varices or clinically significant portal hypertension in patients with hepatocellular carcinoma. Aliment Pharmacol Ther.

[12] Fierbinteanu-Braticevici C, Tribus L, Peagu R (2019). Spleen stiffness as predictor of esophageal varices in cirrhosis of different etiologies. Sci Rep.

[13] Danielsen KV, Hove JD, Nabilou P (2021). Using MR elastography to assess portal hypertension and response to beta-blockers in patients with cirrhosis. Liver Int.

[14] Jangouk P, Turco L, De Oliveira A, Schepis F, Villa E, Garcia-Tsao G (2017). Validating, deconstructing and refining Baveno criteria for ruling out high-risk varices in patients with compensated cirrhosis. Liver Int.

[15] Wu CW, Lui RN, Wong VW (2023). Baveno VII criteria is an accurate risk stratification tool to predict high-risk varices requiring intervention and hepatic events in patients with advanced hepatocellular carcinoma. Cancers (Basel).

[16] Wang P, Hu X, Xie F (2023). Predictive value of liver and spleen stiffness measurement based on two-dimensional shear wave elastography for the portal vein pressure in patients with compensatory viral cirrhosis. PeerJ.

[17] Gao X, Guo XY, Yang LB (2023). Letter to editor 'non-invasive model for predicting high-risk esophageal varices based on liver and spleen stiffness'. World J Hepatol.

[18] Yang LB, Gao X, Li H (2023). Non-invasive model for predicting high-risk esophageal varices based on liver and spleen stiffness. World J Gastroenterol.

[19] Villanueva C, Albillos A, Genescà J (2019). β blockers to prevent decompensation of cirrhosis in patients with clinically significant portal hypertension (PREDESCI): a randomised, double-blind, placebo-controlled, multicentre trial. Lancet.

[20] Parikh F (2016). Infections and thrombocytopenia. J Assoc Physicians India.

[21] Kim SU, Lee JH, Kim DY (2012). Prediction of liver-related events using fibroscan in chronic hepatitis B patients showing advanced liver fibrosis. PLoS One.

[22] Dajti E, Ravaioli F, Marasco G (2022). A combined Baveno VII and spleen stiffness algorithm to improve the noninvasive diagnosis of clinically significant portal hypertension in patients with compensated advanced chronic liver disease. Am J Gastroenterol.

[23] Sharma P, Kirnake V, Tyagi P, Bansal N, Singla V, Kumar A, Arora A (2013). Spleen stiffness in patients with cirrhosis in predicting esophageal varices. Am J Gastroenterol.

